# Older Americans' Attitudes Toward Caregiving Cost Responsibility and Long‐Term Care Access and Costs by Caregiver Status

**DOI:** 10.1111/jgs.70385

**Published:** 2026-03-24

**Authors:** Sarah E. Patterson, John Biziorek, Adriana Reyes, Erica Solway, Matthias Kirch, Dianne C. Singer, Sydney N. Strunk, Jeffrey T. Kullgren, J. Scott Roberts

**Affiliations:** ^1^ Institute for Social Research University of Michigan Ann Arbor Michigan USA; ^2^ School of Medicine University of Michigan Ann Arbor Michigan USA; ^3^ Brooks School of Public Policy Cornell University Ithaca New York USA; ^4^ Institute for Healthcare Policy and Innovation University of Michigan Ann Arbor Michigan USA; ^5^ Center for Clinical Management Research, VA Ann Arbor Healthcare System Ann Arbor Michigan USA; ^6^ Departments of Internal Medicine and Health Management and Policy, Schools of Medicine and Public Health University of Michigan Ann Arbor Michigan USA; ^7^ Department of Health Behavior and Health Equity, School of Public Health University of Michigan Ann Arbor Michigan USA

**Keywords:** attitudes, care costs, caregiving, long‐term care

## Abstract

**Background:**

Rising numbers of older adults will intensify demand for unpaid care from family and friends (caregiving) and quality paid home, assisted living, or nursing home care (long‐term care). With growing public desire for government support, it is important to explore older Americans (age ≥ 50) views, especially by caregiver status.

**Participants and Setting:**

Data were collected from the February–March 2024 wave of the University of Michigan National Poll on Healthy Aging (NPHA), a nationally representative survey of community‐dwelling U.S. adults age ≥ 50 (*n* = 3216).

**Methods:**

We conducted a cross‐sectional study using weighted regression models to examine older Americans' (1) views on who should primarily pay for caregiving (government, family/older adults, or others), (2) concerns about older adults in their community being able to access quality long‐term care, and (3) concerns about long‐term care costs. The key predictor was whether the respondent was a caregiver of an adult ≥ age 65.

**Results:**

Opinions on who should primarily pay for caregiving were evenly split between government (45%) and older adults and their families (45%). Caregivers were less likely to favor older adults and their families bearing primary financial responsibility relative to the government (RRR 0.68, *p* < 0.01). Most older Americans were somewhat or very concerned about quality long‐term care access (80%) and costs (88%), and caregivers were more likely to be concerned about both access (*b* = 1.73, *p* < 0.001) and costs (*b* = 1.44, *p* < 0.01) than noncaregivers.

**Conclusions:**

Most older Americans are concerned about access to long‐term care and costs, yet remain divided on who primarily should pay for caregiving costs. Caregivers are both more concerned about long‐term care access and more likely to support the government's primary responsibility for caregiving costs than noncaregivers. Policymakers should consider more options for access to affordable, high‐quality long‐term care, and financial supports for caregivers.

## Summary

- Key points
  ○ Older Americans' (age ≥ 50) views on who should have primary financial responsibility for covering the cost of caregiving for older adults are evenly split between government (45%) and the older adult and their families (45%).
  ○ Caregivers to an older adult are more likely than noncaregivers to see the government as bearing primary responsibility for caregiving costs (*p* < 0.01).
  ○ Most older Americans are somewhat or very concerned about both costs of (88%) and access to quality long‐term care (home, assisted living, or nursing home care) (80%), and caregivers are more concerned than noncaregivers about long‐term care access (*p* < 0.001) and costs (*p* < 0.01).
- Why does this paper matter?
  ○ This brief report offers a current picture of how U.S. adults age 50 and older, including those providing care to someone age 65 and older, view primary financial responsibility for the overall costs of caregiving of older adults, as well as access and cost challenges for long‐term care (quality home, assisted living, or nursing home care).
  ○ Identifying concerns about caregiving and long‐term care costs provides actionable directions for policymakers to consider expanding supports for affordable, high‐quality long‐term care and target financial support for caregivers.

## Graphical abstract

Most older Americans, age 50 and older, are split between seeing older adults and their families (white) and the government (blue patterned) as primarily responsible for caregiving costs. A greater proportion of caregivers see the government as primarily responsible compared to non‐caregivers. Chi‐square tests show a significant difference in attitudes between caregivers and non‐caregivers regarding primary responsibility for costs (*p* < 0.05).

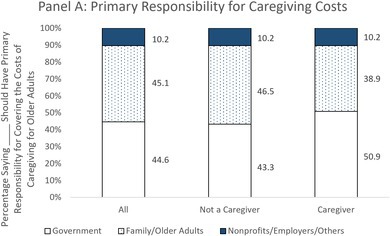

## 1 Introduction

The population of older adults who will need access to unpaid care provided by family and friends (henceforth “caregiving”) as well as high quality paid home, assisted living, or nursing home care (henceforth “long‐term care”) is expected to grow over the next few decades [1, 2]. Currently, a majority (88%) of care for older adults in the U.S. is provided by family [3], but having greater care needs increases the likelihood of using paid services and thus the cost of care [4]. It is projected that seven in 10 adults who survive to age 65 will develop needs for care (i.e., medical and personal care to carry out daily tasks) during their remaining lifetime, and nearly one‐half will ultimately rely on paid care [5]. Often, older adults with care needs require a variety of forms of care in order to meet all of their needs [6].

Both caregiving and long‐term care can be costly. Caregivers spend on average over $7000 per month out of pocket to care for an older adult [7]. Nationally, the annual cost of 8 h of daily paid home health aide services is just over $90,000, whereas a private nursing home room averages more than $115,000 per year [8]. However, older adults and caregivers may have difficulty paying for different forms of care and understanding how to access programs to cover care costs [9]. The infrastructure required to meet this critical need remains costly, and scholars have proposed plans for covering care costs between public programs and individuals, necessitating more research on older adults' attitudes toward caregiving, long‐term care, and their respective costs [6, 10]. Funding for programs that support older adults can impact whether their care needs are met [11].

Recent polls reveal a nearly even split between those who believe the government should pay for older adults' care needs and those who assign primary responsibility to families (broadly defined). However, Americans increasingly favor government support of older adults' care needs, either through providing direct care or covering costs of care [12, 13], or more affordable, expanded eligibility for financial access to care [14]. Further, those closest to these experiences—caregivers of an older adult (age ≥ 65)—may have differing opinions compared to noncaregivers. For instance, caregivers are more likely to support direct payments to caregivers compared to noncaregivers [14].

To better understand older Americans' views toward responsibility for caregiving costs and concerns about the access to and costs of long‐term care, we analyzed nationally representative data from the University of Michigan National Poll on Healthy Aging (NPHA; 2024) [13]. Our objectives were to (1) characterize current opinions among U.S. adults age ≥ 50 about whether government, families/older adults, or other entities should bear primary financial responsibility for caregiving costs to older adults; (2) assess concern among U.S. adults age ≥ 50 regarding both access and costs of long‐term care (defined as quality home, assisted living, and nursing‐home care); and (3) compare these attitudes by caregiver vs. noncaregiver status.

## 2 Data and Methods

We used data from the University of Michigan National Poll on Healthy Aging (NPHA), conducted via NORC's AmeriSpeak panel fielded February–March 2024. The NPHA is a national probability‐based panel of community‐dwelling adults. The study was deemed exempt by the University of Michigan Institutional Review Board, with informed consent waived. The survey completion rate was 43.7% (3379/7731), a similar completion rate found in other online surveys [15]. The final analytic sample was adults ages 50+ with complete cases (3216/3379, 95.2% of the original sample).

### 2.1 Variables

We administered three survey items on care for older adults. First, we measured views on primary caregiving cost responsibility by asking: “Who do you think should have primary responsibility for covering the cost of caregiving for older adults?” Response options were listed as exclusive categories: government; employers; nonprofit organizations; family, relatives, or friends; the person needing care; or other. We collapsed response options to three groups to facilitate comparison to other published studies: “Government” served as a reference, and the relative risk ratios of “Family/Older Adults” and “Private Providers/Others” were calculated. This item was developed to mirror a measure in the General Social Survey (GSS), a nationally representative study of American attitudes, that asks “Who do you think should primarily cover the costs of this help?” (in relation to care provided to an older adult) [16].

The second and third measures focused on access and cost of quality home/assisted living/nursing home care which we refer to as long‐term care. The poll asked respondents “How concerned are you about the following for older adults in your community?” to which they responded to a list of 26 randomized items, including “Access to quality home care/assisted living/nursing home care” and “Cost of home care/assisted living/nursing home care”. Response options were: very concerned; somewhat concerned; or not concerned.

**TABLE 1:** Characteristics of respondents by caregiver status (adults age 50 and older; weighted percentages).

Characteristics	Total sample (*N* = 3216)	Not a caregiver for an older adult (*N* = 2617)	Caregiver for an older adult (*N* = 599)	Sig. diff.
Age				
50–64	54.2	52.8	60.3	*
65–74	27.3	28.3	22.8	
75+	18.6	18.9	16.9	
Sex				
Male	53.0	50.4	64.8	
Female	47.0	49.6	35.2	***
Race and ethnicity				
Asian, Non‐Hispanic	4.3	3.9	6.0	
Black, Non‐Hispanic	10.0	10.1	9.7	
Hispanic	11.6	11.7	11.2	
Other, Non‐Hispanic	3.4	3.6	2.2	
White, Non‐Hispanic	70.7	70.7	70.8	
Marital status				
Married	57.6	55.9	65.9	**
Widowed	8.6	9.3	5.2	
Divorced/Separated	22.4	23.5	17.2	
Never Married	11.4	11.4	11.7	
Any children under 18 in home				
No	89.7	89.4	90.7	
Yes	10.3	10.6	9.3	
Education				
High school or less	40.5	42.2	32.8	**
Some college/Associates	28.4	28.2	29.4	
Bachelor's degree or higher	31.1	29.6	37.8	
Income ($)				
< 25,000	15.9	16.7	12.6	
25–49,999	24.3	24.9	21.4	
50–75,000	18.6	18.1	20.9	
75–149,999	29.3	29.0	30.7	
150,000	11.8	11.2	14.3	
Employment status				
Currently working	44.1	43.1	48.7	
Retired	40.7	41.2	38.8	
Currently not working	15.2	15.8	12.5	
Political ideology				
Liberal	17.4	17.2	18.2	
Moderate/Unknown	48.1	48.1	48.0	
Conservative	34.5	34.6	33.8	
Self‐rated health				
Good, very good, excellent	80.4	79.9	82.7	
Poor or fair	19.6	20.1	17.3	
Daily activities limited due to health problems/disability				
No	62.4	62.7	61.0	
Yes	37.6	37.3	39.0	

*Note:* Data from the National Poll on Healthy Aging (2024). Sig. Diff. = Significant difference between caregivers and noncaregivers using *t*‐test or chi squared tests. *p < 0.05, **p < 0.01, ***p < 0.001.

We drew covariates from NPHA demographic items and focused on caregiver status as the key predictor. Caregiver status was determined by whether the respondent was a caregiver to an adult age 65 or older (yes/no). Other covariates in the model included age, sex, race and Hispanic ethnicity, marital status, any children under 18 in the home, education, income, employment status, political ideology, self‐rated health, daily activities limited due to health problems/disability, and survey mode (phone or internet, controlled but coefficient not shown in tables; see table notes).

#### 2.1.1 Statistical Methods

Models were weighted with NPHA poststratification weights to account for complex design and nonresponse so that estimates reflect a nationally representative sample. We first provided weighted descriptive statistics on the sample, followed by a three‐panel figure that illustrates attitudes for the three main outcomes by caregiver status. We then ran a weighted multinomial logistic regression, controlling for respondent characteristics to estimate who older Americans see as primarily responsible for covering caregiving costs, followed by ordered logistic regressions regarding concerns about long‐term care access and costs. Analyses were performed in Stata 19.5 (StataCorp LLC, College Station, TX).

## 3 Results

**FIGURE 1:**
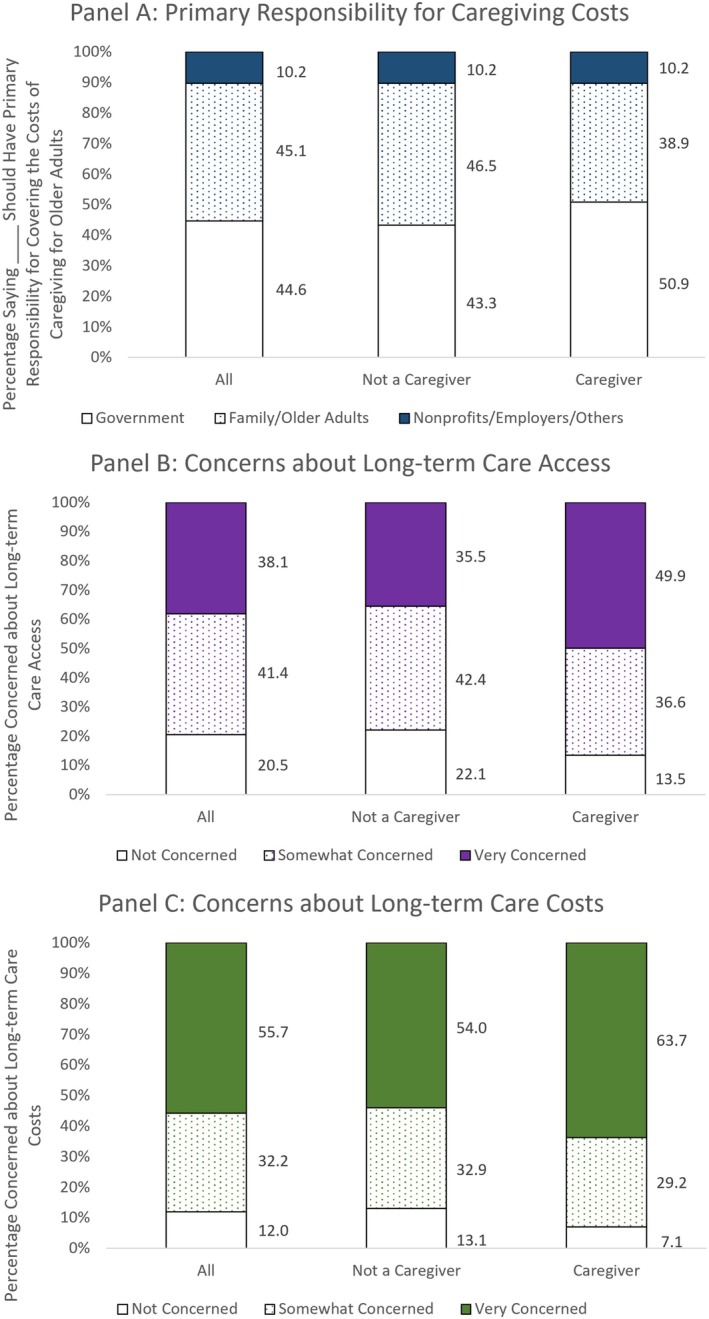
Attitudes toward caregiving cost responsibility and concerns about long‐term care access and costs, by caregiver status. Panel A Legend: Most older Americans are split between seeing older adults and their families (white) and the government (blue patterned) as primarily responsible for caregiving costs. A greater proportion of caregivers see the government as primarily responsible compared to noncaregivers. Chi‐square tests show a significant difference in attitudes between caregivers and noncaregivers regarding primary responsibility for costs (*p* < 0.05). Panel B Legend: Over a third of older Americans are very concerned about older adults' in their community's access to quality long‐term care (purple), and caregivers are more likely than noncaregivers to be very concerned. Chi‐square tests show a significant difference in attitudes between caregivers and noncaregivers regarding access to long‐term care (*p* < 0.001). Panel C Legend: Over half of older Americans are very concerned about older adults' in their community's access to quality long‐term care costs (green), and caregivers are more likely than noncaregivers to be very concerned. Chi‐square tests show a significant difference in attitudes between caregivers and noncaregivers regarding costs of long‐term care (*p* < 0.001).

A majority of older Americans age 50 and older (82.3%) were not providing care to an adult age 65 and older (Table 1). Chi‐square and *t*‐tests show that older adults who were caregivers were more likely to be younger (*p* < 0.05), female (64.8% of caregivers vs. 50.4% of noncaregivers, *p* < 0.001), married (65.9% of caregivers vs. 55.9% of noncaregivers, *p* < 0.01), and have higher levels of education (*p* < 0.01) when compared with noncaregivers. Figure 1, Panel A illustrates bivariate analysis of older Americans' attitudes toward primary responsibility for caregiving costs. While 45.1% of older adults saw the family or older person who needs care as primarily responsible for costs, a similar proportion (44.6%) saw the government as primarily responsible. Caregivers were more likely than noncaregivers to endorse primary government responsibility (50.9% vs. 43.3% respectively, *p* < 0.05). Figure 1, Panel B illustrates older adults' concerns about long‐term care access. While 20.5% were not at all concerned, the majority were either somewhat (38.1%) or very concerned (41.4%). Caregivers were more likely to be very concerned about access to long‐term care compared to noncaregivers (49.9% vs. 35.5%, *p* < 0.001). Figure 1, Panel C illustrates concerns about long‐term care costs. Again, the majority of older adults (87.9%) were somewhat or very concerned (32.2% somewhat, 55.7% very), and caregivers were more likely to be very concerned than noncaregivers (63.7% vs. 54.0%; *p* < 0.001).

Regression analyses showed that these descriptive patterns held even after accounting for demographic characteristics of the respondents (Table 2). Caregivers were less likely than noncaregivers to support family/older adults having primary responsibility for caregiving costs relative to government responsibility (RRR = 0.68, *p* < 0.01). Caregivers had greater log odds of concern about long‐term care access (*b* = 1.73, *p* < 0.001) and costs (*b* = 1.44, *p* < 0.01) compared to noncaregivers. Other covariates were associated with attitudes. Women and Black, non‐Hispanic older adults were less likely to state that they perceived the older adults or their families as primarily responsible for caregiving costs relative to the government; however, adults age 75 and older, those with a Bachelor's degree or higher, those with higher incomes, and political moderates and conservatives were all more likely to state they perceived the older adults and their families as primarily responsible compared to government. Regarding concerns about quality long‐term care access, adults age 75 and older, moderates, and conservatives were less concerned, but women and those in poor/fair health were more concerned. Regarding concerns about quality long‐term care costs, women were more concerned, but moderates and conservatives were less concerned.

**TABLE 2:** Regression analyses for caregiving responsibility and long‐term care access and costs.

	Primary responsibility for caregiving costs: Family/older adults vs. government (multinomial logit)		Primary responsibility for caregiving costs: Nonprofits/others vs. government (multinomial logit)		Concerns about long‐term care access (ordered logit)		Concerns about long‐term care costs (ordered logit)	
	Coeff.	SE	Coeff.	SE	Coeff.	SE	Coeff.	SE
Caregiver to an older adult	0.68**	(0.10)	0.87	(0.19)	1.73***	(0.22)	1.44**	(0.17)
Age								
50–64								
65–74	1.27	(0.18)	1.09	(0.20)	0.95	(0.12)	1.01	(0.13)
75+	1.59*	(0.33)	1.10	(0.32)	0.66**	(0.10)	0.86	(0.15)
Sex								
Male								
Female	0.78*	(0.08)	1.02	(0.19)	1.53***	(0.13)	1.42***	(0.13)
Race and Ethnicity								
White, non‐Hispanic								
Asian or Pacific Islander, non‐Hispanic	1.32	(0.40)	1.13	(0.59)	0.86	(0.24)	0.92	(0.28)
Black, non‐Hispanic	0.60***	(0.08)	0.60*	(0.14)	1.23	(0.13)	0.91	(0.12)
Hispanic	1.14	(0.23)	1.71	(0.48)	1.20	(0.22)	1.13	(0.20)
Other, non‐Hispanic	2.08	(0.84)	2.76*	(1.15)	1.17	(0.35)	0.74	(0.19)
Marital status								
Married								
Widowed	0.96	(0.21)	0.94	(0.30)	0.86	(0.24)	1.01	(0.18)
Divorced/separated	0.98	(0.13)	1.38	(0.31)	1.23	(0.13)	1.04	(0.12)
Never married	1.15	(0.20)	1.02	(0.30)	1.20	(0.22)	0.87	(0.15)
Any children under 18 in home								
No								
Yes	0.74	(0.14)	0.81	(0.26)	1.17	(0.35)	0.94	(0.16)
Education								
High school or less								
Some college/associates	1.19	(0.15)	1.09	(0.23)	0.99	(0.11)	1.15	(0.13)
Bachelor's degree or higher	1.33*	(0.19)	1.20	(0.29)	1.17	(0.16)	1.23	(0.15)
Income ($)								
< 25,000								
25–49,999	1.26	(0.25)	0.86	(0.27)	1.10	(0.20)	1.02	(0.20)
50–75,000	1.73**	(0.35)	0.97	(0.26)	1.20	(0.19)	1.06	(0.20)
75–149,999	1.75**	(0.34)	1.08	(0.30)	1.14	(0.18)	1.03	(0.19)
150,000 >	2.47***	(0.59)	0.62	(0.22)	1.06	(0.20)	0.84	(0.20)
Employment status								
Currently working								
Retired	1.08	(0.16)	1.10	(0.24)	0.89	(0.11)	0.86	(0.11)
Currently not working	0.77	(0.12)	0.96	(0.26)	1.13	(0.17)	0.94	(0.16)
Political ideology								
Liberal								
Moderate/Unknown	2.13***	(0.32)	1.63	(0.41)	0.64***	(0.08)	0.70**	(0.09)
Conservative	3.59***	(0.63)	2.23*	(0.71)	0.51***	(0.07)	0.54***	(0.08)
Self‐rated health								
Good, very good, excellent								
Poor or fair health	0.96	(0.14)	0.72	(0.18)	1.35*	(0.17)	1.09	(0.18)
Daily activities limited due to health problems/disability								
No								
Yes	0.83	(0.12)	1.16	(0.23)	1.17	(0.13)	1.19	(0.13)
Constant	0.27***	(0.08)	0.17***	(0.08)	—	—	—	—
Cut 1	—	—	—	—	0.24***	(0.06)	0.13***	(0.04)
Cut 2	—	—	—	—	1.65	(0.44)	0.77	(0.24)

*Note:* Data from the National Poll on Healthy Aging (2024). Models also control for survey mode (Coefficient not shown). *p < 0.05, **p < 0.01, ***p < 0.001.

Abbreviations: Coeff. = Coefficient; SE = Standard error.

## 4 Discussion

Understanding how Americans age 50 and older view primary responsibility for caregiving costs and access and costs of long‐term care is essential for understanding how policies and programs may be crafted to support both older adults and their caregivers. Older adults were almost evenly split on whether the government versus older adults and their families should have primary responsibility for caregiving costs, replicating similar findings and suggesting a continuing trend [12, 13]. Extending prior studies, we find higher levels of support for the government's primary responsibility among a sample of older adults age 50 and older (44.6%) compared to other studies among older adults ages 65 and older (35%) [12]; this difference suggests that it is younger‐older adults driving these trends. Americans also reported high levels of concern for older adults in their community regarding access to quality long‐term care (including home care, assisted living, nursing home care) and the costs of that care; these rates are similar to levels of concern Americans have for their own access to high quality health care [13].

Building on emerging scholarship, we extend an understanding of these attitudes among caregivers—those who are closest to the experience but not often surveyed separately in relation to their attitudes—relative to noncaregivers [14]. This study contributes new evidence by demonstrating that caregivers to older adults are systemically more likely than noncaregivers to support government responsibility for caregiving costs and report heightened concern about long‐term care access and affordability. These findings are consistent with other recent work showing that caregivers are more likely to support direct payment to caregivers [14]. Caregivers, given their personal experience and potential for greater likelihood of interacting with government programs and formal care systems in their caregiving activities, may be more aware of, and thusly concerned about, the caregiving costs given their own experiences with caregiving and its physical, psychological, and financial consequences [17, 18, 19, 20]. Our results are in contrast, however, to prior findings that have shown no significant difference between caregivers and noncaregivers regarding attitudes toward making long‐term care (home, or in a facility) more affordable. These mixed results across studies suggest that, though both caregivers and noncaregivers have similar levels of agreement on making access more affordable [14], caregivers are especially concerned about those costs. Older adults who have not experienced the need for caregiving or long‐term care may be less able to judge the probability of needing care and how to finance it [21].

This study has limitations. First, the survey was cross‐sectional, so we cannot determine whether heightened concern about long‐term care access or cost, or payment responsibility regarding caregiving translates into future behavior or policy preferences over time. Relatedly, items used general terms such as “care”; future iterations could more clearly specify unpaid vs. paid aspects of care. Second, although NORC's AmeriSpeak panel is probability‐based, the completion rate was low, although similar to other comparable online studies [15]; importantly, NORC oversamples a diverse sample of respondents, and our analyses applied design weights which account for nonresponse bias. Third, the sample excludes adults who reside in assisted living or nursing‐home settings, potentially underestimating concern among families with first‐hand exposure to paid long‐term care. Finally, the poll was conducted in February–March 2024; public attitudes may have shifted as state caregiving reforms advance and media attention to aging policy fluctuates.

While older adults in the U.S. are equally divided between whether older adults and their families or the government should be primarily responsible for covering caregiving costs, taken in context with other work in this area, a growing proportion of Americans are in favor of government support of older adults across time. Providing care to an adult age 65 and older is associated with greater desire for the government to be primarily responsible for caregiving costs, as well as concern about long‐term care access and costs for older adults in one's community. These attitudes signal areas for programs and policymakers to provide more information and supports for older adults, their families and other unpaid caregivers, including home and community‐based services (HCBS) waiver, tax credits for caregivers, and other government programs and policies.

## Author Contributions

Patterson and Reyes conceptualized the study. Solway, Kirch, Singer, Kullgren, and Roberts were responsible for data collection and curation. Patterson, Reyes, and Kirch developed the modeling. Patterson conducted the data analysis and visualization. Patterson, Biziorek, and Reyes wrote the original draft. Patterson, Biziorek, Reyes, Solway, Kirch, Singer, Strunk, Kullgren, and Roberts provided review and editing.

## Funding

This work was supported by the National Institute on Aging of the NIH (K99AG073473, R00AG073473). Support was also provided by the Department of Veterans Affairs, Veterans Health Administration, Health Services Research and Development. The University of Michigan National Poll on Healthy Aging (NPHA) is directed by the University of Michigan Institute for Healthcare Policy and Innovation (IHPI) and receives funding from Michigan Medicine. Data used in this paper were from a wave of the NPHA for which AARP was a sponsor. The content is solely the responsibility of the authors and does not necessarily represent the official views of the NIH, the Department of Veterans Affairs, the United States government, or the authors' employers.

## Disclosure

Sponsors did not have a role in the design, methods, subject recruitment, data collection, analysis, or preparation of the paper.

## Conflicts of Interest

Dr. Kullgren has received grant funding from the US National Institutes of Health, the US Department of Veterans Affairs, the American Diabetes Association, the Robert Wood Johnson Foundation, the Donaghue Foundation, the Healthwell Foundation, the State of Michigan Department of Military and Veterans Affairs, the Michigan Health Endowment Fund, and the Blue Cross Blue Shield of Michigan Foundation. Dr. Kullgren has received consulting fees from SeeChange Health, HealthMine, the Kaiser Permanente Washington Health Research Institute, and the Washington State Office of the Attorney General; and honoraria from the Robert Wood Johnson Foundation, AbilTo Inc., the Kansas City Area Life Sciences Institute, the American Diabetes Association, the Luxembourg National Research Fund, the Donaghue Foundation, the National Science Foundation, the National Institutes of Health, the University of California‐Los Angeles, the University of Pennsylvania, Oak Ridge Associated Universities, and the University of Southern California.
